# Guidelines for the Use and Reporting of Patient-Reported Outcomes in Multiple Myeloma Clinical Trials

**DOI:** 10.3390/cancers15245764

**Published:** 2023-12-08

**Authors:** Edward Laane, Sam Salek, Esther Natalie Oliva, Christine Bennink, Solène Clavreul, Paul G Richardson, Christof Scheid, Katja Weisel, Tatyana Ionova

**Affiliations:** 1 Hematology-Oncology Clinic, Tartu University, 50406 Tartu, Estonia; 2 School of Life and Medical Sciences, University of Hertfordshire, Hatfield AL10 9AB, UK; m.s.salek@herts.ac.uk; 3 U.O.C. Ematologia, Grande Ospedale Metropolitano Bianchi Melacrino Morelli, 89124 Reggio di Calabria, Italy; enoliva@gmail.com; 4 Department of Hematology, Erasmus University Medical Center, 3015 GD Rotterdam, The Netherlands; c.bennink@erasmusmc.nl; 5 Myeloma Patients Europe, 1050 Brussels, Belgium; 6 Jerome Lipper Multiple Myeloma Center, Dana Farber Cancer Institute, Boston, MA 02215, USA; paul_richardson@dfci.harvard.edu; 7 Department of Internal Medicine I, University of Cologne, 50923 Cologne, Germany; c.scheid@uni-koeln.de; 8 Department of Hematology, Oncology and Bone Marrow Transplantation with Section of Pneumology, University Medical Center Hamburg-Eppendorf, 20246 Hamburg, Germany; k.weisel@uke.de; 9 Quality of Life Unit, Saint Petersburg State University Hospital, 190103 Saint Petersburg, Russia; tation16@gmail.com

**Keywords:** multiple myeloma, patient-reported outcomes, quality of life, symptoms, systematic review, clinical trials, guideline

## Abstract

**Simple Summary:**

It is recognized that patients with multiple myeloma (MM) experience a high burden of disease and treatment-related symptoms that impact upon their quality of life (QoL). In these patients, patient-reported outcome (PRO) measures are important in providing information on how treatment affects their QoL. In the past 10 years in the MM setting, the main focus has been to achieve the most durable remission with the best QoL as primary goals of therapy. Optimizing the QoL of patients with MM is an important treatment goal and the use of PROs in clinical trials has the potential to improve treatment outcomes. The present report, on behalf of the European Hematology Association (EHA), provides evidence-based guidelines for the use and reporting of PROs in patients with MM that have been developed according to the EHA’s core Guidelines Development Methodology. Currently, there is considerable variation in the measurement of QoL in MM trials, thus underlining the importance of systematic measurements. These Guidelines will aid clinicians, regulatory agencies and the pharmaceutical industry in the measurement of QoL in patients with MM in clinical trials.

**Abstract:**

In the era of personalized medicine there is an increasing need for the assessment of patient-reported outcomes (PROs) to become a standard of patient care. Patient-reported outcome measures (PROM) are important in assessing significant and meaningful changes as a result of an intervention based on a patient’s own perspective. It is well established that active multiple myeloma (MM) can be characterized by a high burden of disease and treatment-related symptoms, with considerable worsening of quality of life (QoL). In general, and over the past decade, the focus has shifted to obtaining the most durable remissions with the best QoL as primary goals for MM treatment. Patients place considerable value on their QoL and communicating about QoL data prior to treatment decisions allows them to make informed treatment choices. Consequently, optimization of QoL of patients with MM is an important therapeutic goal and the incorporation of PROs into clinical trials has the potential of improving treatment outcomes. In this regard, guidance for the use and reporting of PROMs in MM in clinical trials is warranted. Under the auspices of the European Hematology Association, evidence-based guidelines for the use and reporting of PROs in patients with MM have been developed according to the EHA’s core Guidelines Development Methodology. This document provides general considerations for the choice of PROMs in MM clinical trials as well as a series of recommendations covering a selection of PROMs in MM clinical trials; the mode of administration; timing of assessments; strategies to minimize missing data; sample size calculation; reporting of results; and interpretation of results.

## 1. Introduction

Multiple myeloma (MM) is a malignant neoplasm of plasma cells that accumulates in the bone marrow, leading to renal failure, hypercalcemia, bone destruction, and anemia, as well as other cytopenia due to marrow failure [1]. MM accounts for 1–1.8% of all cancers and is the second most common hematological malignancy, with an estimated incidence in Europe of 4.5–6.0/100,000/y [2]. Among patients with MM, approximately 73% have anemia, 79% osteolytic bone disease, and 19% acute kidney injury at the time of presentation [3]. Impaired immune function is also an important characteristic of the disease that leads to severe infections [4]. In addition, delayed diagnosis has an impact on health, treatment and treatment response [5]. MM is most frequently diagnosed among people aged 65 to 74 years, with the median age being approximately 70 years [6]. Novel treatment approaches have changed the paradigm for MM management. At present, MM is steadily becoming more treatable and manageable with more complex approaches [7]. Partial or complete response with first-line therapy is now highly attainable with minimal residual disease (MRD) negative status also increasingly achievable, particularly for those patients with standard risk disease [8]. The availability of high-dose therapy and novel therapies, such as immunomodulatory drugs, proteasome inhibitors, monoclonal antibodies, other next generation small molecules and immunotherapeutic approaches, has significantly prolonged survival [9]. However, long-term follow-up still reflects a pattern of ongoing relapse, even from long-standing complete response or partial response. Thus, curing, defined as permanent eradication of the myeloma clone, is rarely, if ever achieved. In this context, the focus of attention has evolved to obtaining the most durable remission with the best health-related quality of life (QoL) as primary goals of MM treatment [10]. In this regard, QoL can be considered an important end-point in observational and clinical trials aimed at assessing the efficacy of MM treatment, although measurement has historically been variable. Conversely, the prognostic significance of QoL scores for survival in MM has been shown [11,12,13].

It is well established that MM can be characterized by a high burden of disease and treatment-related symptoms with considerable worsening of QoL, i.e., reduced physical and role functioning, fatigue and pain as the major problems [14,15]. The treatment of MM can be toxic in itself and have a negative impact on patient’s QoL and may be accompanied by multiple side effects [16,17]. Of special note is the importance of evaluating treatment adverse effects, taking into account the patient’s perspective, in addition to objective toxicity assessment [18]. The impact of new treatments on QoL is now an important component of decision making in relapsed MM [19]. Patients want to know what to expect, what adverse events they will face, for how long, how serious these events can be and how long events can last and if they are reversible. More importantly, understanding how the treatment will affect their everyday life and the activities they usually/want to undertake is key. It has been shown that up to half of study participants’ symptomatic adverse events in clinical trials are not reported, leading to underestimation of potential harm [20]. Therefore, the optimization of the QoL of patients with MM can be considered an important treatment goal and the incorporation of patient-reported outcomes (PROs) into clinical trials has the potential for improving treatment outcomes [21,22,23].

Patient-reported outcome measures (PROMs) are important in assessing significant and meaningful changes as a result of an intervention based on a patient’s own perspective. The two main categories of PROMs are ‘disease-specific’ and ‘generic’. Disease-specific PROs are focused on the impact and the symptoms of the respective disease and because of this inherent aspect are more sensitive to measuring change over time. However, they lack depth and breadth in terms of the wider aspects of daily activities, in particular those of a psychosocial nature. On the other hand, area (therapeutic)-specific generic PROMs, such as those that are hematology-specific, are wider in scope and coverage and allow comparisons between different conditions within the same therapeutic area. In recent years, there has been increasing use of “symptom” or intervention-specific PRO tools, such as those measuring the impact of peripheral neuropathy and those measuring the impact of transplantation.

Consideration and reporting of the PROMs’ minimal clinically important difference or change (MCID, also referred to as MID and MIC) when interpreting change over time in the context of clinical trials is of paramount importance. The MCID is the smallest change in outcome that patients consider important, thereby justifying a change in patient management or claim of a therapeutic effect of an intervention. Therefore, this aspect of PRO reporting is particularly important in the interpretation of PROM scores.

### Medical Products Regulatory Authorities’ PRO Guidelines

In cases where PROMs are used in clinical trials of medical products to support labeling claims, this guideline should be consulted in conjunction with the following PRO guidelines:

(a) The US FDA 2009 PRO Guidelines [24].
(b) The US FDA 2021 Core PROs in Cancer Trials Guidelines [25].
(c) The EMA 2006 Reflection Paper on the use of health-related QoL measures [26].
(d) The EMA 2015 Reflection Paper on the use of PRO measures in oncology studies [27].

In recent years, the US FDA has granted PRO labeling to very few oncology medicines. The FDA and the EMA use different levels of evidence for assessing PRO data from oncology trials. It appears that the EMA based their decision largely on open-label studies, on broad concepts such a QoL and PRO measures. However, this approach may not be suitable for current haemato-oncology clinical trials. It is important to appreciate that there are key differences in the levels of evidence used by the FDA and the EMA and therefore an understanding of these differences may be useful to guide PRO measurement strategies as pharmaceutical companies or investigators intend to use PROs for labeling purposes. The addition of a QoL assessment may also be useful if pursuing labeling from the EMA [28]. It is important to note that some HTA bodies utilize QoL in their modelling. For example, NICE in the UK uses the QALY [29]. The EQ-5D tools, despite their obvious limitations, are important to include in clinical trials for reimbursement purposes in some countries.

## 2. Methods

### 2.1. EHA Core Guidelines Development Methodology

Under the auspices of the European Hematology Association (EHA), the development of guidelines for the use and reporting of PROs in clinical trials in adult patients with MM was conceptualized following the EHA core Guidelines Development Methodology, as summarized below.

#### 2.1.1. Appointment of Chair(s) and Steering Committee Selection 

The Chair for this project was appointed by the EHA Guidelines Committee (EHA-GC) together with partner EHA Scientific Groups/patient organizations/network in cooperative initiatives. A Steering Committee comprising 3 members was subsequently selected by the Chair with the specific responsibility of developing guidelines. The Guidelines Committee reviewed disclosure of any potential conflicts of interest prior to officially appointing these positions. In cooperative initiatives, the Chair agreed on the general project objectives, methodology and conflict-of-interest policy to be adopted. 

#### 2.1.2. Selection of an Expert Panel

The Steering Committee selected an Expert Panel comprising members experienced in the area of MM with interest in PROs that were both active in clinical care as well as research. The selection also took into consideration geographical representation and specific areas of expertise. The EHA-GC reviewed the disclosure of potential conflicts of interest of the Members of the Panel. The Expert Panel assumed the responsibility of the detailed definition of the aim of the project and development of main clinical questions, the synthesis of the scientific evidence and the formulation of recommendations. The Expert Panel incorporated whenever possible patient representatives of the corresponding disease area for the definition of objectives and final review.

#### 2.1.3. Handling of Conflicts of Interest

The Steering Committee and the Expert Panel (including all potential experts involved) were obliged to state any financial interests or affiliations, providing information on specific institutions, organizations/companies and competing interests that may be perceived as bias in the present guideline. Prior to accepting the official appointment, all individuals involved (the Chair, the Steering Committee, the Expert Panel and all potential experts) were requested to disclose an actual, potential or perceived conflict of interest with regard to their relationship with the pharmaceutical industry and medical professional organizations, in addition to their involvement in developing other guidelines. During the execution of the project there was no reasonable perception of a conflict of interest. 

#### 2.1.4. Definition of the Objective of the Project and Generation of Key Questions 

A first Panel meeting (web-based video conference) was organized to define the objectives of the project and formulate key questions specifically addressing the appropriate development process and psychometric testing strategies, candidate patient groups/sub-groups and risks derived from the use of the PRO instrument(s) in clinical trials. The list of questions was rank-ordered and then selected using the criterion for clinical research use within the context of repeated measurements. 

#### 2.1.5. Systematic Literature Review and Synthesis of Evidence 

Members of the Expert Panel were assigned a specific issue, based on the previously defined set of key questions and according to the member’s specific area of interest. Each member was invited to undertake a literature review on the assigned issue and to prepare a summary of the available evidence relevant to the key questions that were under consideration. To support the panelists, the selection, collection and review of the articles of interest was undertaken by a dedicated person with access to literature sources and expertise in the appraisal of scientific evidence. The literature search was undertaken according to the following criteria (in addition to other criteria specific to the topic under consideration):

(a) English language;
(b) Publication year: 2015 to 2020 (literature search period undertaken from 1 January 2015 to April 2021);
(c) Clinical studies, including the target population (patients with MM);
(d) Source: PubMed, proceedings of EHA and ASH meetings.

An evidence table was then prepared, presenting summaries of studies relevant to each of the key clinical questions addressed by the guideline, and the level of evidence was rated using the robustness of the psychometric testing for the respective PRO’s measurement properties. GRADE (Grades of Recommendation Assessment, Development and Evaluation) or SIGN (Scottish Intercollegiate Guidelines Network) or equivalent systems were considered not relevant for the development of this type of guideline. The quality of evidence for each of the main outcomes was determined according to the study design, study quality, consistency and directness and ranked accordingly. 

#### 2.1.6. Consensus Phase 

Members of the Expert Panel independently formulated evidence-based statements to address the key questions. Furthermore, two consensus meetings of the Expert Panel were organized to reach a consensus of the synthesis of evidence for all of the recommendations for the use and reporting of PROs in MM clinical trials. 

### 2.2. Systematic Review

The Cosmin guideline for Systematic Reviews for PROMs [30] was followed. PROMs frequently used in MM patients in randomized clinical trials from 2015 to 2020 and their psychometric properties were reviewed.

The study design, disease and treatment characteristics, primary outcome and the implemented PRO instrument(s) were extracted using a pre-defined template. To evaluate the consistency of PRO reporting, study registries were compared with publications, study protocol and Health Technology Assessment (HTA) reports, whenever available.

Overall, 10,707 records were identified, in which 38 different PRO instruments were reported. Finally, 118 studies were selected as appropriate for review [31]. Subsequently, publications after April 2021 were reviewed by the two independent reviewers for their registration on clinicaltrial.gov before 2020 as the systematic review strategy included RCTs registered on clinicaltrial.gov. This search did not reveal any new or relevant material, rendering the information presented here up-to-date.

Other reports and systematic reviews on the use of PROs in MM clinical trials have also been considered [32,33,34,35,36,37,38].

## 3. Results

### 3.1. Evidence-Based Consensus on the Choice of Patient-Reported Outcome Measures

#### 3.1.1. General Considerations

The instrument of choice should possess robust measurement properties. It should meet the following requirements: validity, reliability, and sensitivity to change in MM, with a published MCID. 

To analyze and to maximize the utility of submitted PRO information, it is recommended that the core PROs, namely disease-related symptoms, symptomatic adverse events, overall side effect impact summary measure, physical function and role function, according to recent FDA Guidance for the pharmaceutical industry [25], are to be collected.

The choice of PROM(s) in clinical trials depends on the combination of the following factors:

(a) Disease status;
(b) Primary and secondary objectives;
(c) In the case of multinational trials, the availability of translations (linguistic and cross-cultural adaptations).

The measures that have been identified through the systematic literature review as appropriate are listed in Table 1 and Table 2. Characteristics of PROMs not recommended for MM, identified by systematic review, are shown in Supplementary Table S1.

#### 3.1.2. Generic PROMs

Amongst generic PROMs, such as SF-36 and all variants of European Quality of Life-5 Dimensions (EQ-5D) scales, the only one fulfilling the requirements of the systematic literature review was the EQ-5D-3L. 

##### EQ-5D-3L

(a) General information: The EQ-5D-3L is a generic measure designed to assess health status both in specific patient groups as well as the general population [39,40]. It was developed by the EuroQol group as a standardized instrument for describing and valuing QoL and to inform evidence on the cost-effectiveness of alternative treatments.

The EQ-5D is a preference-based QoL measure with one question for each of the five dimensions (mobility, self-care, usual activities, pain/discomfort and anxiety/depression). Answers given to the EQ-5D enable the possibility to find 243 unique health states or can be converted into the EQ-5D index. This is a utility score anchored at 0 for death and 1 representing perfect health. The EQ-5D questionnaire also includes a Visual Analog Scale (VAS), in which patients can report their perceived health status with a grade ranging from 0 (representing worst possible health status) to 100 (representing best possible health status). The instrument can also be used to calculate cost-effectiveness in healthcare (e.g., Quality Adjusted Life Years; QALYs).

(b) Data collection: Paper and electronic.
(c) Mode of administration: Self-administration.
(d) Median completion time: 9 min [41].
(e) Measurement properties for MM: Sensitivity to change has been demonstrated in MM [42]. MCID reported for MM patients [42].
(f) Strengths: Simplicity. It provides a simple, descriptive profile and an overall numeric estimate of QoL which can be used for both clinical and economic evaluations of health care. It is available in over 80 languages and its wide use in clinical trials in a range of various health conditions and treatments allows for multiple comparisons. Information about MCID in MM has been published.
(g) Weaknesses: Validation in MM patients is needed. The EQ-5D-3L, a utility measure, does not satisfy the requirements of the FDA Guidance for core PROMs in clinical trials [25]. No equivalence studies between electronic and paper versions are available.

#### 3.1.3. Cancer-Specific PROMs

The only PROM fulfilling the requirements of the systematic literature review was the EORTC QLQ-C30.

##### EORTC QLQ-C30

(a) General information: The EORTC QLQ-C30 is a QoL questionnaire that was developed by the European Organization for Research and Treatment of Cancer (EORTC) Quality of Life Study Group to measure QoL in cancer patients during clinical trials [43]. This questionnaire consists of thirty items, five function scales (physical, emotional, social, role and cognitive); three symptom scales (fatigue, nausea/vomiting and pain); and six single items (dyspnea, insomnia, appetite loss, constipation, diarrhea and financial difficulties). The two latter items are used to assess global health and overall QoL. The majority of item responses are on a four-point scale ranging from one (not at all) to four (very much), with a recall period of one week. The two items assessing global health and overall QoL have a response option of seven categories ranging from one (very poor) to seven (excellent).
(b) Data collection: Paper and electronic.
(c) Mode of administration: Self-administration.
(d) Completion time: <10 min [44].
(e) Measurement properties in MM: Validated in a sample of patients with newly diagnosed MM [45]. Its reliability, validity and sensitivity to change have been demonstrated [45]. MCID reported for MM patients [32].
(f) Strengths: It is easy to use and simple to score. It captures core PROs identified by the FDA Guidance, disease-related and treatment-related cancer symptoms, symptomatic adverse events, physical function and role function [25]. It is available in over 110 languages and has been widely used in clinical trials in MM and several other malignancies, allowing for comparisons. Information about MCID in MM is published.
(g) Weaknesses: An additional questionnaire or the EORTC QLQ-MY20 module is still needed to cover specific MM issues. No equivalence studies between electronic and paper versions are available. It worth noting that it is also quite old as a PRO tool so may not adequately cover some of the side-effects of newer agents like chimeric antigen receptor T-cell (CAR-T).

#### 3.1.4. Disease-Specific PROMs

##### EORTC QLQ-MY20

(a) General information: The EORTC QLQ-MY20 was developed by the EORTC Study Group on Quality of Life (1999) and is an instrument specifically designed for MM patients [46]. It includes twenty disease-specific items, consisting of three multi-item scales (disease symptoms; six items, side effects of treatment; ten items, future perspective; three items) and body image; single item scale. It is developed for use in conjunction with the EORTC QLQ-C30 in MM patients varying in disease stage and treatment modality.
(b) Data collection: Paper.
(c) Mode of administration: Self-administration.
(d) Completion time: 12 min [46].
(e) Measurement properties: Validated in a sample of MM patients in an international study [46]. Its reliability, validity and sensitivity to change have been demonstrated [47]. Anchor-based MCIDs are reported [47].
(f) Strengths: It is simple to score and easy to use. The instrument is available in 50 languages. Information about MCID is published.
(g) Weaknesses: The focus is on the frequency of symptoms which MM patients may experience, not on their severity. It is worth noting that it is also quite old as a PRO tool so may not adequately cover some of the side-effects of newer agents like CAR-T. Its use in conjunction with the EORTC QLQ-C30 may render the self-report burdensome.

##### FACT-MM

(a) General information: FACT-MM was developed with the aim to create an MM-specific PROM as part of the FACT measurement system to assess the MM-related disturbances [48,49]. The FACT-MM questionnaire includes 41 items consisting of four core FACT-G QoL subscales (physical, functional, social and emotional comprising 27 items) and an additional subscale (MM subscale) measuring MM-specific concerns (comprising 14 items). After following standard FACT instructions, items are rated using a 0–4-point scale based on the past week, with higher scores indicating better QoL and less MM-related symptoms. Three scores are calculated: the FACT-MM total score (all domains; scale, 0–164), the trial outcome index (TOI; physical, functional and MM-specific domains; scale, 0–112) and an MM subscale score (scale, 0–56).
(b) Data collection: Paper and electronic.
(c) Mode of administration: Self-administration and interview when applicable.
(d) Completion time: 10 to 15 min [50].
(e) Measurement properties: Validated in patients with newly diagnosed MM [48]. Its reliability, validity and sensitivity to change have been demonstrated [49]. MCIDs are based on patient-reported assessment of meaningful change, defined as 10% of the instrument range and on mean baseline differences between ECOG groups (0 vs. 1 to 2) [51].
(f) Strengths: Captures core PROs outlined by the FDA; disease-related and treatment-related cancer symptoms and physical function [25]. The instrument is available in nine languages. Information about MCID is published.
(g) Weaknesses: No equivalence studies between electronic and paper versions are available.

##### MDASI-MM

(a) General information: The MD Anderson Symptom Inventory for Multiple Myeloma (MDASI-MM) questionnaire was developed in the MD Anderson Cancer Center to assess the severity of the symptoms related to MM and its treatment and the impact of these symptoms on daily functioning [52]. The MDASI-MM questionnaire includes the MDASI’s thirteen core symptom severity items and six interference items [53], in addition to seven MM-specific items (bone aches, muscle weakness, sore mouth/throat, rash, difficulty concentrating, constipation, diarrhea). Patients rate their symptoms on a 0–10 scale, ranging from “not present” to “as bad as you can imagine”. Interference is rated on a 0–10 scale ranging from “did not interfere” to “interfered completely”. MDASI-MM ratings can be used to derive three subscale scores: mean core (thirteen MDASI core symptom items), mean severity (thirteen MDASI core plus seven MM-specific items), and mean interference (six interference items). Interference items may be subdivided into mean activity-related (interference with work, general activity and walking ability—WAW) and mean mood-related (interference with relations with people, enjoyment of life and mood—REM) dimensions [52]. Symptom severity is classified as 0 (none), 1–4 (mild), 5–6 (moderate) or 7–10 (severe) [52].
(b) Data collection: Paper and electronic.
(c) Mode of administration: Self-administration and interview or via telephone-based interactive voice response (IVR) system.
(d) Completion time: 5 min [53].
(e) Measurement properties: Validated in a sample of MM patients [53]. Its reliability, validity and sensitivity to change have been demonstrated [53]. MCIDs are not reported.
(f) Strengths: Comprehensive yet concise tool that is recommended as a uniform symptom assessment instrument for patients with MM in research and practice. The instrument is available in over 30 languages.
(g) Weaknesses: No information about MCIDs is available. No equivalence studies between electronic and paper versions are available. Also, there is not enough perspective from MM studies.

#### 3.1.5. Other QoL Instruments

There are several instruments that have been developed in recent years applying both traditional as well as sophisticated techniques for PROM development. However, they have not yet undergone testing in clinical trials. Such measures are often referred to as the new generation of PROMs and, hence, show the importance of being mentioned in these guidelines. 

##### MyPOS

(a) General information: The MyPOS is the first myeloma-specific QOL questionnaire designed specifically for use in clinical practice [54]. It consists of thirty items in three scales: symptoms and function (fourteen items), emotional response (six items) and healthcare support (five items). The MyPOS is based on qualitative enquiry and the issues most important to MM patients. It focuses on the impact of symptoms rather than the status of MM symptoms.
(b) Data collection: Paper.
(c) Mode of administration: Self-administration.
(d) Completion time: 7 min [54].
(e) Measurement properties: Validated by the authors in a large sample of MM patients with different treatment status (on- and off-treatment), different disease phase (newly diagnosed, stable/plateau phase, relapsed/progressive), and in various settings (hospital inpatient, outpatient, at home). Its reliability, validity and sensitivity to change have been demonstrated [54]. Information about MCIDs is lacking.
(f) Strengths: It is a brief and comprehensive tool.
(g) Weaknesses: It has not been tested in clinical trials. No information about MCIDs. Available only in two languages (English and German).

##### HM-PRO

(a) General information: HM-PRO is the only hematological malignancy (HM)-specific PROM [55,56]. It evaluates QoL and symptoms of patients with HM and has been developed directly from the experience of patients for patients. It consists of two scales: Part A (24 items) measuring the ‘impact on patients’ QoL; and Part B (18 items) measuring ‘signs and symptoms’ (S&S) experienced by the patients. Part A consists of four domains: physical behavior (seven items), social well-being (three items), emotional behavior (eleven items) and eating and drinking habits (three items). Higher scores represent higher impact.
(b) Data collection: Paper and electronic.
(c) Mode of administration: Self-administration.
(d) Completion time: 7 min [57].
(e) Measurement properties: Validated in samples of patients with hematological malignancies, including MM. Its reliability, validity (content, construct and discriminant) and sensitivity to change have been demonstrated [58,59].
(f) Strengths: A comprehensive tool that measures the impact of the disease and treatment, including stem cell transplantation, on QoL and signs and symptoms in patients with HM. PROMs between different HMs may be compared by the same instrument. The instrument is available in 11 languages. Electronic and paper version equivalence [57] as well as MCID [60] have been reported.
(g) Weaknesses: There are no published reports yet about the use of HM-PRO in clinical trials.

##### FACT-BMT

(a) General information: The FACT-BMT is the Bone Marrow Transplant subscale of the FACT-G [61]. It includes 23 items specifically to assess QoL and symptoms in bone marrow transplant patients and, along with 27 general questions, it consists overall of 50 items. Patients rate all items using a five-point scale, ranging from 0—not at all to 5—very much. They are asked to rate themselves on how they feel today and have felt over the past 7 days. A higher score indicates better QoL or less symptoms. The FACT-BMT provides information about overall QoL and the dimensions of physical well-being, social/family well-being, emotional well-being, functional well-being and transplantation-specific concerns.
(b) Data collection: Paper and electronic.
(c) Mode of administration: Self-administration and interview when applicable.
(d) Completion time: 10–15 min [62].
(e) Measurement properties: Validated in a sample of leukemia patients in China. Its reliability, validity and sensitivity to change have been demonstrated [63]. MCIDs are not reported in MM patients.
(f) Strengths: The questionnaire is able to cover transplantation-specific concerns in MM patients undergoing hematopoietic stem cell transplantation (HSCT). The instrument is available in 40 languages.
(g) Weaknesses: There are no separate modules for autologous and allogeneic patients despite the different experiences of these types of transplant patients. No information about MCID and sensitivity to change in MM patients. No equivalence studies between electronic and paper versions are available.

#### 3.1.6. Symptom Scales

The majority of MM patients experience numerous symptoms, such as pain, fatigue and psychological disturbances, therefore different tools may be recommended to assess symptoms. To measure the severity of a single symptom, visual analogue scales (VAS) and numerical rating scales (NRS) are utilized. Symptom assessment measures are also available.

(1) Pain assessment

Pain is a prevalent symptom of MM. However, it is not possible to recommend a specific PROM due to lack of evidence. Therefore, pain in MM patients may be assessed using pain-specific scales.

(2) Fatigue Assessment

The FACIT Fatigue Scale is the only PROM which has been reported for the assessment of fatigue in MM patients. 

##### FACIT Fatigue Scale

(a) General information: The FACIT Fatigue Scale is a 40-item measure that assesses self-reported fatigue and its impact upon daily activities and function [64]. It includes thirteen items specifically designed to test fatigue and the FACT-G Physical well-being (seven items), Social/Family well-being (seven items), Emotional well-being (six items) and Functional well-being (seven items). Patients rate all items using a five-point scale ranging from 0—not at all to 5—very much. The recall period is 7 days.
(b) Data collection: Paper and electronic.
(c) Mode of administration: Self-administration and interview when applicable.
(d) Completion time: 10–15 min [65].
(e) Measurement properties: Validated in a sample of patients with chronic lymphocytic leukemia [66]. Its reliability, validity and sensitivity to change have been demonstrated [66].
(f) Strengths: The questionnaire is able to measure fatigue and its impact upon daily activities and function in MM patients. The instrument is available in over 60 languages. Fatigue in MM may be compared to fatigue in other malignancies measured by the same instrument which has been widely used in clinical trials.
(g) Weaknesses: Information about MCID is lacking. No equivalence studies between electronic and paper versions are available and MCIDs are not reported.
(3) Assessment of Psychological Disturbance

The Hospital Anxiety and Depression Scale (HAD Scale) is the most frequently reported for the assessment of psychological disturbance in MM patients. 

##### HAD Scale

(a) General information: The HAD Scale is a 14-item PROM which has been found to perform well in assessing symptom severity, anxiety and depression in somatic, psychiatric and primary care patients, as well as in the general population [67]. The recall period is one week. Even-numbered questions relate to depression and odd-numbered questions relate to anxiety. Each question has four possible responses on a scale from 0 to 3. The maximum score for each domain, depression and anxiety, is 21. A score of 8–10 is suggestive of the presence a mood disorder, whereas a score of at least 11 indicates the probable presence of a mood disorder. The two domains have been found to be independent measures. In its current form, the HAD Scale is now divided into four ranges: normal (0–7), mild (8–10), moderate (11–15), and severe (16–21).
(b) Data collection: Paper and electronic.
(c) Mode of administration: Self-administration.
(d) Completion time: 2–5 min [67,68].
(e) Measurement properties: Validated in a sample of patients with hematologic malignancies [69]. Its reliability, validity and sensitivity to change have been demonstrated [70].
(f) Strengths: It is sensitive to changes both during the disease and in response to psychotherapeutic and psychopharmacological intervention [70]. Additionally, HAD Scale scores can be used to predict psychosocial and possibly physical outcomes as well. It is available in 127 languages.
(g) Weaknesses: No equivalence studies between electronic and paper versions are available. MCIDs are not reported in MM patients.

#### 3.1.7. Other Instruments

To assess safety in a clinical trial, the Patient-Reported Outcomes version of the Common Terminology Criteria for Adverse Events (PRO-CTCAE) is a library of 124 items that measures 78 symptomatic adverse events designed for eliciting patient-reported adverse events in oncology [71]. It is being adopted across clinical trials and regulatory agencies to promote its adoption and the development of standardized PRO-CTCAE analyses and reporting. Each item is analyzed individually. Therefore, algorithms are being developed to generate a single composite numerical grade for each PRO-CTCAE symptomatic adverse event based on the mapping of its individual item scores and to evaluate the composite grades to assure that their validity, reliability and sensitivity to change are comparable with individual item scores. At the present time, it is recommended to select specific PRO-CTCAE items for a given trial based on common and expected reactions [72]. 

### 3.2. Key Points and Recommendations

In myeloma trials, PROs have been mainly used in addition to toxicity data to prove that more efficacious regimens have no negative impact. In newly diagnosed MM, progression-free survival and, recently, MRD-negativity are typically primary endpoints in clinical trials for first-line treatments. PROs have a value as a primary objective to test regimens with a similar efficacy but different toxicity. With more and more MRD-negative remissions, especially after CAR-T therapy, PROs can support efficacy results in contrast to toxicity and, as such, should be included as a secondary objective that is important to assess benefit/risk balance. 

#### 3.2.1. Selection of the PROMs

PROMs should be selected according to the objectives of the study, the investigational product and the target population, as well as the availability of the culturally adapted translations according to the countries involved in the trial. In MM, the stage of the disease should also be considered when selecting the appropriate instrument.

(a) Primary Objective: Progression-Free Survival and MRD Negativity

The benefit on survival should be supported by PROMs in a way that the positive effect is not affected by a negative impact of the investigational product on PROs. The PROs, in fact, must show stability or, at best, an improvement. 

Appropriate PROMs for the evaluation of PROs in a survival trial are myeloma- or HM-specific PROMs, validated in MM (Table 1). An additional symptom assessment tool may be considered (Table 2).

(b) Primary Objective: QoL and/or Control of Symptoms

The chosen PROM must be myeloma- or HM-specific with a published MCID in MM or previously published reports on changes in PRO scores within clinical trials in the target population (Table 1). Exploring sample sizes among candidate PROMs may establish the best choice according to the feasibility, i.e., multicenter trials may include a larger sample size compared to single-center trials (please also refer to the sample size calculation recommendation below).

In trials that include hematopoietic stem cell transplantation, the tools that can be recommended at present should include FACT-BMT and HM-PRO, with the first being transplant-specific and the latter validated in both MM and transplant patients. Other additional symptom assessment tools may be considered (Table 2). 

#### 3.2.2. Mode of Administration 

PROMs are self-reported in that the patient responds about oneself as an expert on one’s own experience. Patients may complete PROMs by writing (ink) or by electronic computer- or telephone-based platforms. The limitations of written responses are the risk of data entry errors and the time dedicated to data entry, which is overcome by electronic entry. The latter may have limitations for patients uncomfortable with technology, such as elderly patients. It is also recommended that the two versions, electronic and paper, be approved and validated for equivalence by the authors of the PROM. 

It is important that the patient completes the PROM in a quiet and private setting prior to the visit in order to avoid distraction and interreference. Possible settings are upon arrival to the clinic appointment, feasible with both electronic (app) or paper methods, allowing for real-time assessment, only if interruption, anxiety, staff burden and negative impact on clinic flow be avoided. It is ideal to complete the PROM at home on the same day of the first trial visit occurring if compliance is not an issue. 

Another limitation of self-reporting is the inability to respond because of general health conditions or cognitive or communication deficits. These patients are not usually included in clinical trials but may develop such limitations during the trial, accounting for missing data upon subsequent visits. Proxy reports (PROMs completed by someone else) are not used nor recommended in clinical trials, unless specified, because they may not be accurate to represent subjective experiences. In cases in which the PROM is complex or in patients with reading, writing or visual difficulties, an adequately trained interviewer may read questions aloud and record the response. Limitations are the interviewer-associated costs and possible bias related to the interview, social desirability or acquiescent response set [73]. To overcome these limitations, standardized training is recommended for interviews in each participating center. 

#### 3.2.3. Timing of Assessments

At present, the minimum is at least four times: at baseline, at the first visit for the evaluation of safety, at the time-point of the assessment of the primary objective and at the time of disease progression/end of study. 

Additional assessments should be included at study completion or at the time of early discontinuation, when possible. Ideally, assessments of PROMs should be implemented at each trial visit that evaluates the primary endpoint, restricted to an interval of at least 4 weeks (to avoid recall or training effect). An additional assessment may be considered at the screening period for those PROMs that have not enough information on the measurement properties.

All evaluations should be assessed prior to the first trial visit occurring and before receiving the treatment, ideally upon arrival to the clinic appointment.

##### Newly Diagnosed, Treatment-Naive MM

In patients with newly diagnosed MM entering clinical trials to receive first-line treatment, the timing of the PROM assessment must consider the primary and secondary objectives.

(a) Objective: Progression-Free Survival and/or MRD Negativity

The minimum recommended time points (TPs) of the PRO assessment are TP-1 at baseline, TP-2 (first safety assessment evaluation) at the first visit date ≥ 4 weeks from baseline, TP-3 at trial visit that evaluates the primary endpoint, TP-4 at disease progression or study completion or early termination if it does not coincide with TP-3. Additional TPs are recommended after induction, consolidation and during maintenance therapy.

(b) Objective: Response Rate and Overall Survival

The minimum recommended TPs of the PRO assessment are TP-1 at baseline, TP-2 (first safety assessment evaluation) at the first visit date ≥ 4 weeks from baseline, TP-3 at trial visit that evaluates the primary endpoint, TP-4 at disease progression or study completion or early termination if it does not coincide with TP-3. Additional TPs are recommended at intermediate visits and during the follow-up period within the trial to develop a trajectory of QoL and symptoms over time.

(c) Objective: Control of symptoms

Control of symptoms in newly diagnosed MM is not generally a primary end-point, but it can be an important secondary end-point. The minimum recommended TPs of the PRO assessment are TP-1 at baseline, TP-2 at trial visit that evaluates the primary endpoint, TP-3 at disease progression or study completion or early termination if it does not coincide with TP-2. Additional TPs are recommended at intermediate visits and during the follow-up period within the trial [74].

(d) Objective: Safety

New investigational products and procedures are being assessed by safety outcomes as a primary endpoint. The minimum recommended TPs of the PRO assessment are TP-1 at baseline and assessments at every trial-directed clinical visit with at least 4-week intervals. 

##### Relapsed/Refractory MM

In addition to the recommendations given for newly diagnosed MM, further considerations must be made for relapsed/refractory MM. Primary objectives are generally overall response rate and safety. Furthermore, CAR-T therapy represents a novel approach to cancer treatment, particularly advanced cancer. A recent review has reported that clinical trials for CAR-T therapies have failed to collect valuable QoL data [75]. However, newer trial data in 2022 have produced useful results and appropriate PRO instruments and assessments have been conducted at monthly TPs, as recommended [35,36]. 

##### HSCT and CAR-T

In trials that include HSCT.

(a) Trials designed to receive HSCT after the investigational drug is initiated and HSCT are not the investigational procedure per se:

Suggested minimum recommended TPs of the PRO assessment are TP-1 at baseline, TP-2 (first safety assessment evaluation) at the first trial visit date occurring ≥ 4 weeks from baseline, TP-3 at day 0 of HSCT (this may precede TP-3), TP-4 at trial visit that evaluates the primary endpoint and TP-5 at disease progression, study completion or early termination if it does not coincide with TP-3 or TP4. Additional TPs are recommended at intermediate visits and during the follow-up period.

(b) Trials Designed to Receive HSCT as the Investigational Procedure

The minimum recommended TPs of the PRO assessment are TP-1 at day 0 of HSCT, TP-2 at the trial visit that evaluates the primary endpoint, TP-3 at disease progression, study completion or early termination if it does not coincide with TP-2. Additional TPs are recommended at intermediate visits and during the follow-up period.

##### In Trials That Include CAR-T

Suggested minimum recommended TPs of the PRO assessment are TP-1 at baseline, TP-2 (first safety assessment evaluation) at the first visit date occurring ≥ 4 weeks from baseline, TP-3 at the trial visit that evaluates the primary endpoint, TP-4 at disease progression, study completion or early termination if it does not coincide with TP-3 or TP4. Additional TPs are recommended at intermediate visits and during the follow-up period.

#### 3.2.4. Strategies to Minimize Missing Data

Missing data may have an impact on the generation of scales. Generally, if more than 50% of items in a scale are missing, the scale cannot be generated [76]. Thus, it may be important, when considering group trial data, to impute missing data. It is recommended to follow the authors’ instructions for the handling of missing items for each PROM.

(a) Systematic missing data. One of the strategies to reduce the probability of systematic missing items (the patient does not respond voluntarily) is the choice of the PROM which must be relevant to the target population. The PROM should also have gone through proper cultural adaptation in the different languages and countries to ensure the relevance of the PROM.
(b) Random missing data. For paper versions, to minimize missing data, the trial staff members should check the items completed by the patient at the time of administration/submission to encourage to complete all random missing data. For electronic versions, there are several strategies to ensure the completion of the questionnaire, such as restriction to proceed without filling in the appropriate answer; however, the possibility to select “no response” for unwillingness to respond to the item should be avoided.

#### 3.2.5. Sample Size Calculation

To meet the PRO endpoint, proper sample size calculation is recommended.

(a) PRO as a primary endpoint. The PROM requires MCID or previously published changes within a clinical trial. When an MCID is available for the chosen PROM, the sample size may be easily calculated according to the MCID value predicted from baseline to target time point in the study arm/s. In the absence of an MCID, a search in the literature of the changes in the PROM measure scale can inform about the expected minimal difference between arms or between time points.
(b) PRO as a secondary endpoint. The sample size is obtained from the primary endpoint. To reach the secondary endpoint, however, a sample size can be calculated as described above for the PRO as a primary endpoint and the larger of the two sample sizes generated should represent the final sample size for the trial, when feasible. If the sample size cannot be obtained for the PRO endpoint because information is lacking, consider changing the PROM or proceeding with the sample size for the primary endpoint.
(c) PRO as an exploratory endpoint. A sample size calculation is not required.

#### 3.2.6. Reporting Results and Interpretation

The following guidelines on reporting PROM results and their interpretation may be applied to all hematological malignancies, including MM. The systematic publication of PRO results, as well as including these in lay summary results of clinical trials, is recommended so that patients can access easily information on the QoL impacts of the investigated treatments. Additionally, the incorporation of PRO results in registries/big data platforms is recommended.

When reporting results, it is recommended to follow the 2013 CONSORT-PRO extension [77,78,79,80]. The core reporting should include:

(1) Reports with details of study settings, genders, ethnicity and mean and range of participants’ age.
(2) A clear statement of the sample size calculation, randomization and blinding methods, including allocation concealment.
(3) Correct baseline characteristics and comparisons of subjects presented.
(4) Patient numbers, whether intention to treat or per protocol analysis was implemented and the method(s) for data imputation of missing data.
(5) PROM baseline and final data collection point mean and median scores with interquartile ranges, as well as score differences.
(6) The actual scores in the results, which may also be accompanied by percentage score changes.
(7) PRO results using MCID and score severity bands.

The following recommendations have been selected by the authors to be implemented in the reporting and interpretation of results in MM trials.

(a) Stratification According to Baseline PRO Scores

Stratification according to baseline scores may be considered for trials that include PRO changes as a secondary end point in subjects with a relevant proportion expected to have good PRO scores at the baseline, and thus are not in a condition to have a significant improvement (i.e., not reaching the MCID). The stratification should consist in good baseline scores versus poor, the thresholds of which are either provided by the PROM itself by the authors or are otherwise determined by group-based methods which provide a range of thresholds of the trial sample population [81]. Results should then be reported according to the baseline scores.

(b) Changes in PRO Scores

A change in scores from the baseline and between arms by correlations should be reported [82]. Changes in scores should be evaluated with the primary endpoint by the appropriate statistical tests. 

In MM, when the primary endpoint is survival and has been reached, the changes in PRO measures should evaluate the impact of treatment (adverse events, changes in symptoms, changes in quality of life) and consider at least the stability of the PRO measures as a positive effect. When the primary endpoint is a change in a biochemical measure (e.g., hemoglobin level), correlations should be performed with the PRO scores and reported. The perception of the impact of the biochemical change is important to define the efficacy of a given investigational product; therefore, the improvement of the PRO scores is desired.

All domain correlations, independent of statistical significance, should be reported. It is worth noting that correlations may achieve statistical significance but not reach the MCID [82].

(c) MCID

A change in scores from the baseline and between arms is best supported by MCID, generally provided by the PRO measure. The MCID value represents the cut-off value (above and below) to distinguish subjects experiencing a significant change in PRO scores. 

If the PROM does not provide a pre-established MCID value, it may be generated after all the subjects have been included in the trial based on the baseline PRO score. A common formula to calculate the MCID is the following: MCID = 1.96 × √2 × SEM (standard error of measurement) of the baseline domain score [83]. 

## 4. Conclusions

In the era of more and more efficacious therapy options in MM, there is an increasing need for the assessment of PROs to become a standard of patient care. With the increasing interest in the use of PROs in clinical trials, coupled with the complexity of novel treatments for MM impacting survival and patient experience, these in turn can lead to improving the successful translation of clinical trial findings to real world practice [84]. Thus, the development of EHA-directed Guidelines for the use and reporting of PROs is timely. Furthermore, it was envisaged that guidance must be evidence-based to provide stakeholders with confidence to adopt these Guidelines.

When conceptualizing and designing clinical trials, the inclusion of PROMs should be evaluated with this guidance in mind. There is currently wide variation in the measurement of QoL in MM trials, so systematic measurement is important, as is the optimal use of existing tools. This is vital to objectively understand the impact on patient QoL and these data are also important for regulatory as well as HTA decision making. It is therefore recommended that researchers/research organizations should involve patients and patient advocacy organizations, regulators and reimbursement bodies (e.g., via scientific advice) to ensure that their incorporation of the PROM strategy is the correct fit for this purpose. In sum, it is hoped that clinicians, regulatory agencies and our partners in the pharmaceutical industry will find these Guidelines useful for clinical trials in MM.

## Figures and Tables

**Table 1:** Characteristics of QoL measures recommended for MM.

PROM	Characteristics			MCID	Validated In			
	Items, n.	Completion Time	Paper + Electronic		HM	MM	BMT	Languages
EQ-5D-3L	5	<10 min	yes	yes	yes	yes	no	>150
EORTC QLQC30	30	<10 min	yes	yes	yes	yes	yes	110
EORTC MY20 *	20	12 min	yes	yes	no	yes	yes	50
FACT-MM (BMT) *	41	10–15 min	no	yes	yes	yes	yes	9
MDASI-MM *	26	5 min	yes	no	no	yes	no	30
MyPOS *	30	7 min	no	no	no	yes	no	2
HM-PRO **	42	7 min	yes	yes	yes	yes	yes	11

* MM-specific PROM, ** HM-specific PROM, BMT = bone marrow transplantation, EORTC QLQC30 = European Organisation for Research and Treatment of Cancer Core Quality of Life 30-item questionnaire, EORTC MY20 = European Organisation for Research and Treatment of Cancer Quality of Life Multiple Myeloma (20-item) Questionnaire, EQ-5D-3L = European Quality of Life 5 Dimensions 3 Level Version, FACT-MM (BMT) = Functional Assessment of Cancer Therapy quality of life measurement system—multiple myeloma (Bone Marrow Transplant), HM = hematological malignancy, HM-PRO = hematological malignancy (HM)-specific patient-reported outcome measure, MCID = minimal clinically important difference, MDASI-MM = The MD Anderson Symptom Inventory for multiple myeloma, MyPOS = Myeloma Patient Outcome Scale, PROM = patient-reported outcome measure.

**Table 2:** Characteristics of symptom measures recommended for MM.

PROM	Characteristics				Validated In				Symptoms
	Items, n.	Completion Time	Paper + Electronic	MCID	HM	MM	BMT	Languages, n	
NRS/VAS	1	<5 min							Pain
FACIT-F	40	10–15 min	yes	no	yes	no	no	>50	Fatigue
HAD	14	2–5 min	yes	no	yes	no	no	127	Psychological distress
HM-PRO SS	18	7 min	yes	yes	yes	yes	yes	11	Multiple

BMT = bone marrow transplantation, FACIT-F = Functional Assessment of Cancer Therapy—Fatigue, HAD = Hospital Anxiety and Depression, HM = hematological malignancy, HM-PRO SS = hematological malignancy-specific patient-reported outcome measure signs and symptoms, MCID = minimal clinically important difference, MM = multiple myeloma, NRS/VAS = numerical rating scale/visual analogue scale.

## Data Availability

Data are contained within this article.

## Supplementary Materials

The following supporting information can be downloaded at: https://www.mdpi.com/article/10.3390/cancers15245764/s1, Supplementary Table S1: Characteristics of PROMs not recommended for MM, identified by SR.
