# Supplementary material for: Guidelines for the Use and Reporting of Patient-Reported Outcomes in Multiple Myeloma Clinical Trials
Source: Cancers (Basel). 2023 Dec 8;15(24):5764. doi: 10.3390/cancers15245764 (PMC10741926; doi:10.3390/cancers15245764)
Supplement: Supplementary file 1 [file cancers-15-05764-s001.zip › cancers-2731061-supplementary.pdf]

Supplementary Material Table S1. Characteristics of PROMs not recommended for MM, identified by SR.

PROM	Validity	Reliability	Sensitivity to change	MCID
EORTC QLQ MY24	√	√	x	x
SF-36	x	x	x	x
EQ5D-5L	x	x	x	x
FACT G	x	x	x	x
PROMIS (anxiety)	x	x	x	x
PROMIS (depression)	x	x	x	x
PROMIS (fatigue)	x	x	x	x
PROMIS (pain interference)	x	x	x	x
PROMIS (sleep disturbance)	x	x	x	x
PROMIS (physical functioning)	x	x	x	x
PRO-CTCAE	x	x	x	x
FACT BMT	x	x	x	x
FACIT-Sp	x	x	x	x
BPI-SF	x	x	x	x
CTSQ	x	x	x	x
FACT GOG-Ntx	x	x	x	x
LANSS	x	x	x	x
IPAQ-SF	x	x	x	x
PSF-R	x	x	x	x
ASK-12 scores	x	x	x	x
BARS	x	x	x	x
CASE Medication Adherence Index	x	x	x	x
ESES	x	x	x	x
FACT-BMT Trial Outcome Index	x	x	x	x
FACT – Neurotoxicity	x	x	x	x
FACT-Ntx TOI	x	x	x	x
Patient reported convenience and satisfaction with the carfilzomib dosing schedule	x	x	x	x
PGIS	x	x	x	x
PROMIS tool for self-efficacy	x	x	x	x
System Usability Score	x	x	x	x
AQoL-6D	x	x	x	x
MySIm-Q Scale Sore	x	x	x	x

x – unavailable data; √ - available data; R = systematic review
